# Effect of Absorber Placement and Sound Source Type on Room-Acoustic Parameters in a Small Listening Room

**DOI:** 10.3390/ma19214513

**Published:** 2026-10-27

**Authors:** Bartlomiej Chojnacki

**Affiliations:** Department of Mechanics and Vibroacoustics, AGH University of Krakow, 30-059 Krakow, Poland; bchojnacki@agh.edu.pl

**Keywords:** small-room acoustics, sound absorption, room impulse response, source radiation characteristics, reverberation time, early reflections, room acoustic measurements

## Abstract

The acoustic effect produced by a sound-absorbing material in a small room is not determined by laboratory material characterization and installed area alone; it also depends on spatial deployment and on how the room is excited. This study investigates the combined effects of absorbing-material placement and excitation-source characteristics in a full-scale small listening room. Twelve fixed-amount treatment configurations, each using 12 identical polyurethane-foam absorbers with a total nominal area of $6\;m^{2}$, were compared with an untreated reference. Measurements were performed using a quasi-omnidirectional Brüel & Kjær OmniPower 4292-L source and two directional loudspeakers, Genelec 8020D and AudioSolutions O302B Overture, in a fixed stereo-like source–listener geometry. Room impulse responses were evaluated in one-third-octave bands from 63 to 5000 Hz using $T_{20}$ and $C_{80}$. The frequency-resolved results show substantial placement-dependent changes, with the strongest differences occurring mainly in the low and low-mid frequency ranges. As secondary descriptive summaries, the full-band mean $T_{20}$ reduction reached approximately 0.33 s and the mean $C_{80}$ increase approximately 5.9 dB, depending on source and configuration. The relative ordering of treatment configurations was itself source dependent. A representative time-domain comparison further showed source-dependent modification of early reflected energy, whereas later energy was reduced more consistently. The results demonstrate that laboratory material characterization, spatial deployment, source–listener geometry, and excitation conditions should be considered jointly when evaluating acoustic treatment in small listening rooms.

## 1. Introduction

Porous sound-absorbing materials are among the most widely used passive methods of controlling reflected sound energy and decay in enclosed spaces. Their acoustic performance can be characterized under standardized laboratory conditions, but the room-level effect obtained after installation is not determined by laboratory characterization and exposed area alone. Once an absorber is introduced into a room, its effective contribution depends on the sound field incident on that surface and therefore on how the material is coupled to the room geometry, source, receiver, and dominant propagation paths. Identical or comparable absorbing areas can produce measurably different room responses when redistributed between surfaces or moved within the same enclosure [1,2]. This distinction between laboratory material characterization and installed in situ performance is particularly important in small listening environments, where the assumption of a spatially uniform sound field is weak.

Small listening rooms constitute a specific acoustic case because the modal and transition-frequency regions cover a substantial part of the frequency range relevant to reproduction and assessment. In these regions, the field is strongly non-diffuse and spatially heterogeneous: room modes, boundary damping, and the positions of the source and receiver can produce large local differences in sound pressure and decay behavior [3,4,5]. The decay observed in a one-third-octave band may be governed by several resonant contributions with different damping rates, so conventional reverberation descriptors at low frequency should be interpreted as practical decay measures rather than as direct equivalents of statistical reverberation in a diffuse field [6,7]. For treatment design, this means that the effectiveness of a given absorber is expected to depend on whether it is placed where the local sound field couples efficiently to the relevant modes and reflection paths.

The importance of absorber location has been demonstrated in several complementary lines of research. Numerical and experimental studies have shown that changing the position of the same or a comparable amount of absorption can alter low-frequency response, decay, spatial uniformity, and room-acoustic parameters [8,9,10]. Lau and Powell analysed a realistically sized small rectangular room and showed that both the amount and placement of absorption influence the low-frequency sound field and the spatial variation of frequency response [10]. In dwelling layouts, Song and Kang reported a strong dependence of low-frequency attenuation on the room and location in which absorption was introduced [11,12]. Full-scale studies in classrooms and meeting rooms further demonstrated that redistributing absorbing material changes reverberation- and clarity-related metrics, and that the most effective placement depends on the degree of diffusion and the functional use of the room [2,13]. Similarly, Berardi et al. investigated the optimization of sound-absorbing material quantity and placement in large reverberant classrooms using room-acoustic simulations to achieve specified acoustic performance criteria [14]. These studies establish that placement itself is acoustically relevant; however, they do not resolve how practical placement strategies interact with the excitation conditions of a full-scale critical-listening room. The remaining knowledge gap is still present in the terms of small room acoustic when the fixed position of electroacoustic sound source is utilized.

**Figure 1:**
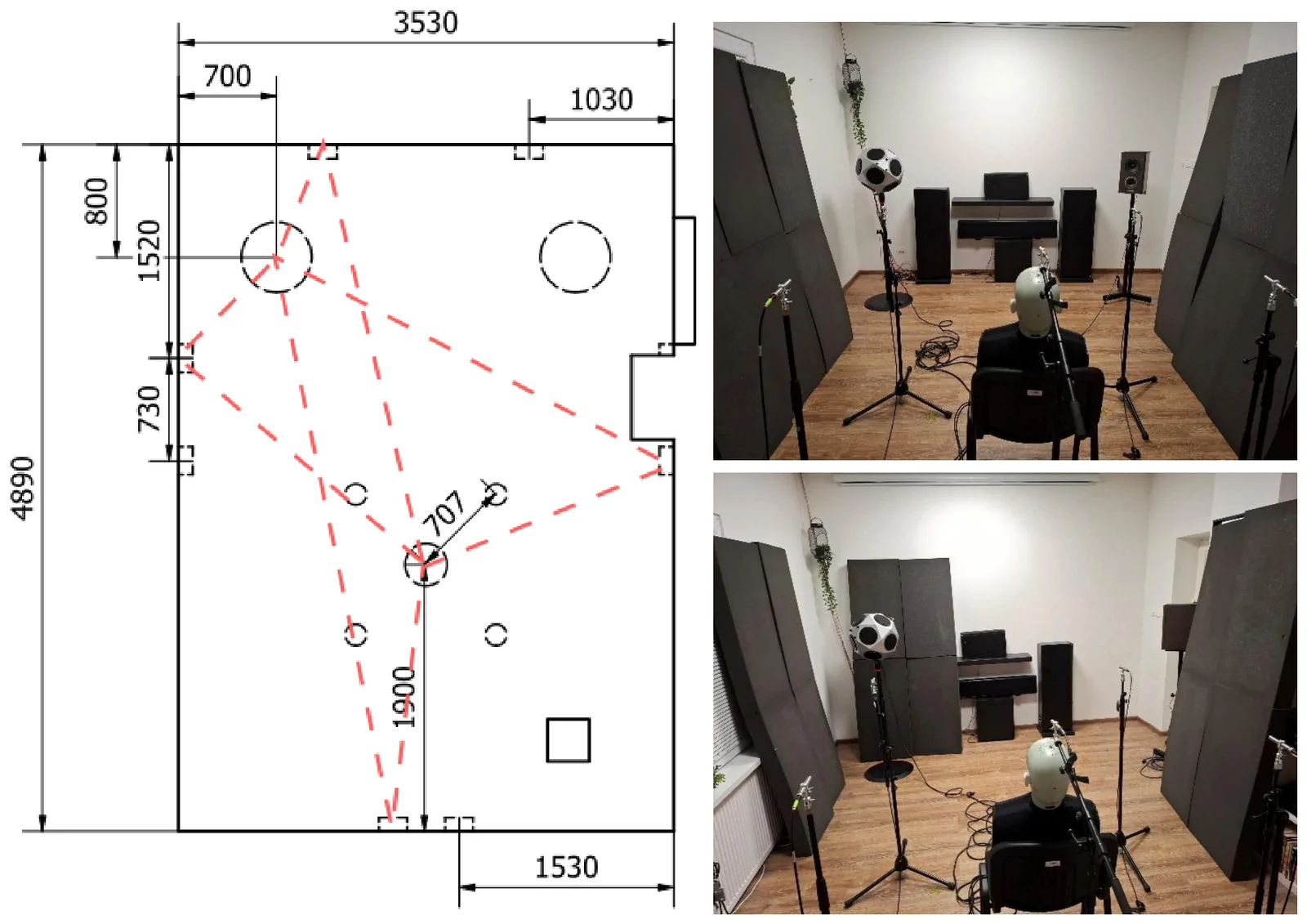
Measurement room and fixed source–listener geometry. (**Left**) plan view showing the two nominal source positions, the central listening region, measurement microphones, and geometrically determined first-order reflection paths from the left source (red dashed lines). (**Right**) photographs of the experimental setup illustrating the sound sources, receiver arrangement, and acoustic treatment.

A listening room differs functionally from rooms in which acoustic quality is characterized mainly through a broadly averaged field. In stereo or near-stereo playback, the source positions are prescribed by the reproduction geometry and the listener occupies a constrained region rather than an arbitrary position in the enclosure. The front, rear, and lateral boundaries therefore have distinct roles with respect to direct sound and early-reflection paths, and treatment at nominally equal surface areas can interact differently with those paths. This source–listener relationship is especially important for first-reflection treatment, where absorber placement is defined relative to a specific propagation path rather than simply by the percentage of room surface covered. Small-room research has long emphasized the importance of source and receiver position, while placement studies indicate that local treatment can modify both the modal field and the frequency response at listening positions [3,10]. Evaluating absorbing-material placement in a critical-listening room requires a measurement strategy that preserves the functional relationship between the loudspeakers, the listener, and the treated surfaces. The experimental geometry and the corresponding first-reflection paths are illustrated in Figure 1.

The sound source used to excite the room introduces an additional methodological factor. Room-acoustic measurements commonly employ multi-element sources designed to approximate omnidirectional radiation, whereas actual listening conditions are produced by directional loudspeakers whose radiation patterns vary with frequency. Practical omnidirectional sources themselves depart from ideal omnidirectionality as frequency increases, and previous work has shown that source radiation and orientation can modify measured room impulse responses and derived acoustic parameters [15,16,17,18,19]. Measurements comparing different omnidirectional-source constructions also demonstrate that source radiation characteristics remain a relevant part of the measurement chain [20]. An omnidirectional measurement source is therefore not intrinsically inappropriate; rather, it represents a different room-excitation condition from a real playback loudspeaker. In a small room, where the field is strongly position- and frequency-dependent, this difference may also change the measured effectiveness of a given absorber arrangement.

The absorber-placement and source-radiation problems have largely been investigated as separate questions. Existing literature provides strong evidence that absorber location affects the acoustic response and, independently, that source radiation affects measured room responses. However, there is limited full-scale evidence combining a fixed amount of the same absorbing material, multiple practical treatment strategies, a fixed stereo-like source–listener geometry, and different source radiation characteristics within the same small listening room. In particular, it remains unclear whether the relative effect of moving a fixed absorber area between front, rear, lateral, first-reflection, and corner regions is preserved when the room is excited by a quasi-omnidirectional measurement source instead of real playback loudspeakers. This combined source × placement dependence is the principal focus of the present work, rather than the already established general observation that absorber location can matter [2,10,16,20].

The aim of this study is therefore to quantify how the spatial deployment of the same sound-absorbing material, defined relative to a fixed stereo-like source–listener geometry, affects selected room-acoustic parameters in a full-scale small listening room, and to determine how these placement-related effects depend on the radiation characteristics of the sound source used for excitation. The analysis addresses four connected questions: (1) how strongly redistribution of a fixed absorber area changes decay time ($T_{20}$) and clarity ($C_{80}$); (2) which controlled changes in treated surface, longitudinal position on the lateral walls, and first-reflection strategy produce consistent differences in the measured room response; (3) whether the relative acoustic effect of a given placement remains the same for a quasi-omnidirectional source and real directional loudspeakers; and (4) how the source–placement relationship varies with frequency and with the early/late temporal structure of the measured room impulse response.

## 2. Materials and Methods

### 2.1. Test Room and Fixed Source–Listener Geometry

Measurements were performed in a furnished laboratory room 5E/D1 at AGH University of Krakow with internal dimensions of approximately 4.89×3.53×2.76m. The room represents a full-scale small listening environment in which the source and receiver positions were fixed throughout the measurement campaign. The experimental geometry was organised around two source positions corresponding to the left and right channels of a stereo-like reproduction arrangement and a central listening region. The source–listener arrangement was designed with reference to the listening geometry described in EBU Tech 3276 [3,21]. The dimensional arrangement used in the experiment is shown in Figure 1, together with the geometrically determined first-reflection paths originating from the left source position. All sound sources were positioned at a height of 1.2 m and directed toward the listening region in the playback-source measurements. When exchanging sound sources, each device was carefully aligned with the corresponding nominal source position, with a positioning accuracy of approximately 5 mm.

The receiver system consisted of five G.R.A.S. 46AE free-field 1/2-inch measurement microphones (Krakow, Poland) and a Brüel & Kjær 4128-C head-and-torso simulator (HATS) (Holte, Denmark). Four G.R.A.S. microphones were distributed around the HATS at a height of 1.2 m, whereas the fifth microphone was positioned approximately 0.10 m above the HATS. The HATS was located in the centre of the listening region. Importantly, all five measurement microphones remained physically fixed at their original positions throughout the entire measurement campaign, without repositioning between absorber configurations or sound sources. This procedure was adopted to ensure consistent receiver geometry and to eliminate intentional microphone-position changes as a source of variation in the measured acoustic parameters, particularly the position-sensitive $C_{80}$. A single operator remained seated at the same location during all measurements, preserving the same occupancy condition throughout the measurement campaign. The measurements were performed at approximately 23 °C and 41% relative humidity; variations in both quantities remained within 5% of these values over the complete campaign and should not disturb the performed measurements [22,23].

The five G.R.A.S. microphones constituted the receiver set used for the primary $T_{20}$ and $C_{80}$ analyses. The two HATS ear channels were excluded from all spectral and scalar averages because their directional weighting is not equivalent to that of free-field measurement microphones. HATS data were retained only for the representative time-domain reflectogram analysis described in Section 2.6. The use of several measurement-microphone positions was particularly relevant in the investigated small room, where the sound field is spatially non-uniform and strongly dependent on both source and receiver positions [3].

### 2.2. Absorbing Material and Placement Configurations

The acoustic treatment consisted of identical absorbers made of acoustic polyurethane foam. Each element had nominal dimensions of 1.00×0.50m and a thickness of 100 mm. The absorption properties of the PU foam is determined by the following parameters [24,25]: the density was approximately $28\;\mathrm{kg}/m^{3}$, the airflow resistivity was approximately $28,000\;\mathrm{Pa}\;s/m^{2}$, and the porosity was approximately 0.98. These material parameters had been determined in previous characterization measurements of the same foam [26,27]. Configurations W1–W12 each contained 12 identical absorbers, corresponding to a nominal exposed absorber area of $6\;m^{2}$, whereas W0 represented the untreated reference condition. Material type, absorber dimensions, and total material quantity were held constant throughout the analysed treatment set. All absorbers were installed flat against the corresponding room boundary, with no deliberately introduced air gap. The interpretation of laboratory absorption data also requires consideration of specimen geometry and measurement conditions, as previous studies have shown that manufacturing imperfections and test-sample configuration can influence the agreement between predicted and measured absorption characteristics [28,29,30].

**Figure 2:**
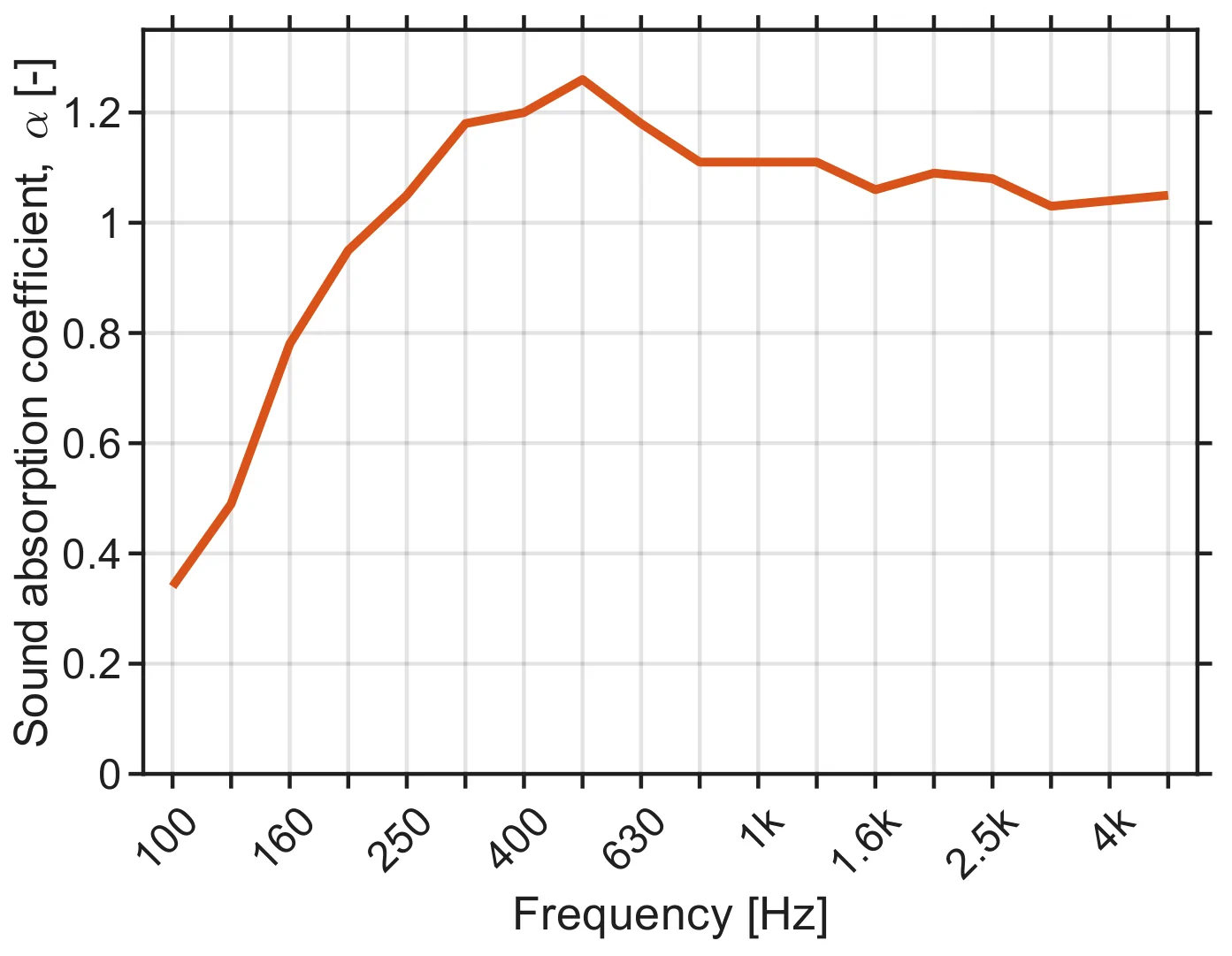
Laboratory-measured frequency-dependent reverberation-room sound absorption coefficient $\alpha_{s}\left(f\right)$ of the 100-mm-thick acoustic polyurethane foam used in all absorber configurations, determined according to ISO 354.

The frequency-dependent sound absorption characteristic of the polyurethane foam used in the experiment is shown in Figure 2. Sound-absorption measurements were conducted in the reverberation chamber at AGH University according to ISO 354 [31]. The resulting reverberation-room absorption coefficient $\alpha_{s}$ provides a laboratory characterization of the absorber and is distinguished here from its room-level effect after installation. Values of $\alpha_{s}$ exceeding 1.0 are a well-known phenomenon in reverberation-room measurements of finite specimens, primarily associated with edge diffraction effects [32,33,34]. Such values do not imply absorption of more than 100% of the incident acoustic energy. The possibility of absorption exceeding 1.0 is also explained in ISO 354 standard. The laboratory characteristic remained unchanged between configurations, whereas the effective in-room utilization of the material depended additionally on its spatial position within the room sound field.

The experimental design therefore distinguishes laboratory material characterization from effective utilization after installation in a particular sound field. Changing the position of the same absorber does not change the measured laboratory absorption characteristic, but it can modify the absorber’s contribution to the room response because different surfaces and regions interact differently with the acoustic field [1,2,10].

The analysed configurations were divided into three groups according to experimental purpose. Configurations W1–W5 investigated treatment of different wall surfaces and different longitudinal positions. In W1 all absorbers were placed on the front wall, whereas in W2 they were placed on the rear wall. Configurations W3–W5 distributed six absorbers on each of the two lateral walls, with the treatment positioned respectively in the front part of the room (W3), the middle part (W4), and the rear part (W5).

Configurations W6–W8 investigated reflection-oriented treatment strategies. W6 combined treatment of the front-wall and lateral first-reflection regions, W7 combined treatment of the rear-wall and lateral regions, and W8 treated the front and rear walls without the corresponding lateral-wall treatment. The first-reflection locations were determined geometrically using the image-source method implemented with the MATLAB acousticRoomResponse workflow for the fixed source–listener geometry [35].

**Figure 3:**
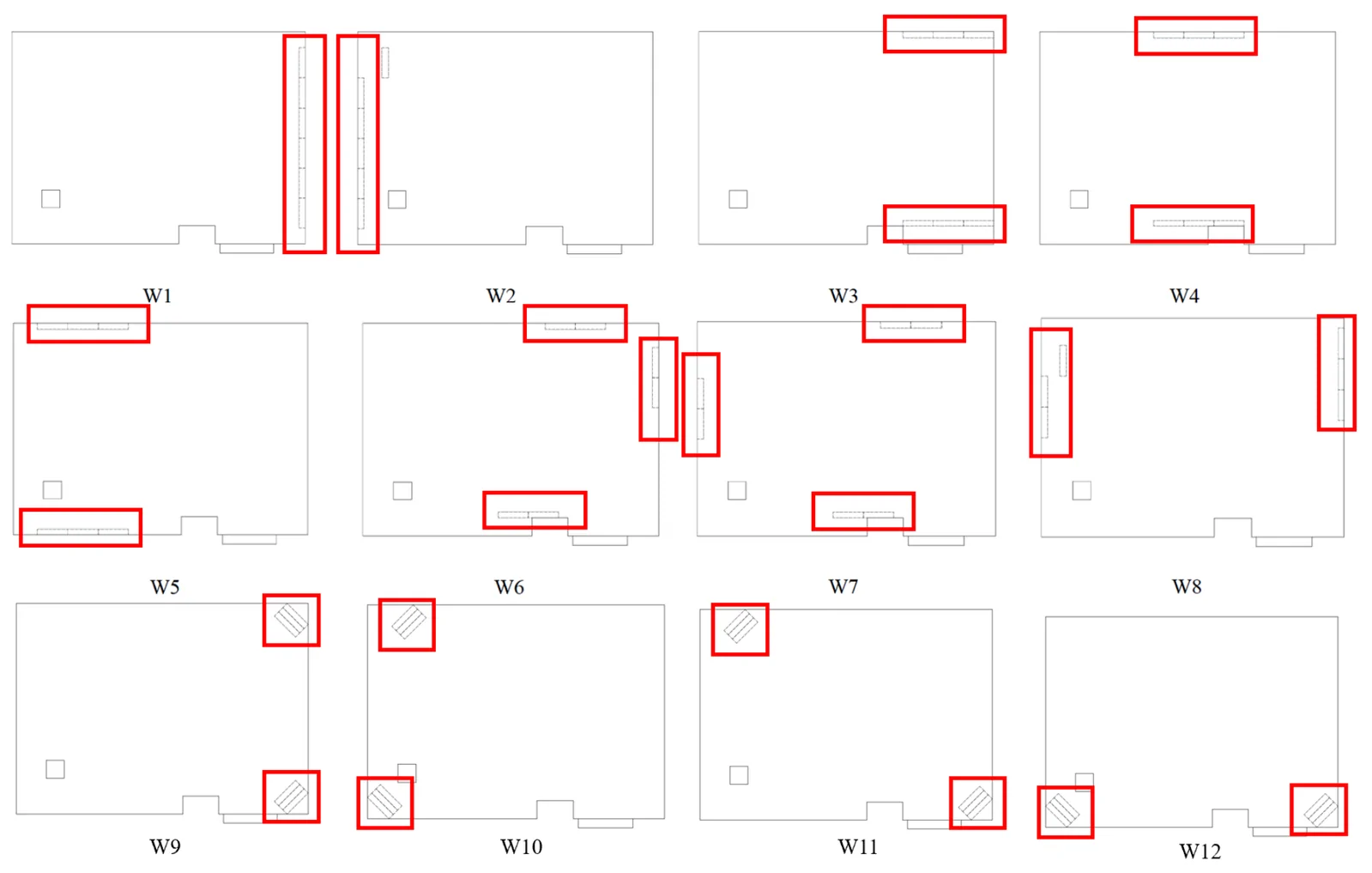
Analysed fixed-amount absorber layouts W1–W12. Red rectangles indicate the treated regions.

Configurations W9–W12 investigated different placements of the same amount of material in room corners: two front corners (W9), two rear corners (W10), diagonally opposite front and rear corners (W11), or a front and rear corner located on the same side of the room (W12). The complete set of analysed treatment layouts is shown in Figure 3.

### 2.3. Sound Sources and Radiation Characterisation

Three sound sources were used to provide deliberately different room-excitation conditions. The first source was a Brüel & Kjær OmniPower 4292-L (Holte, Denmark), representing a measurement-oriented quasi-omnidirectional excitation. The remaining two sources were real directional playback loudspeakers: a Genelec 8020D studio monitor (Warsaw, Poland) and an AudioSolutions O302B Overture loudspeaker (Gliwice, Poland). Each device was successively placed in the same two nominal source positions shown in Figure 1.

The purpose of using these three devices was not to identify one source type as intrinsically preferable for room-acoustic measurements. Instead, source type was introduced as an experimental variable to determine whether the acoustic effect attributed to a given absorber arrangement remains unchanged when the room is excited by devices with different radiation characteristics.

**Figure 4:**
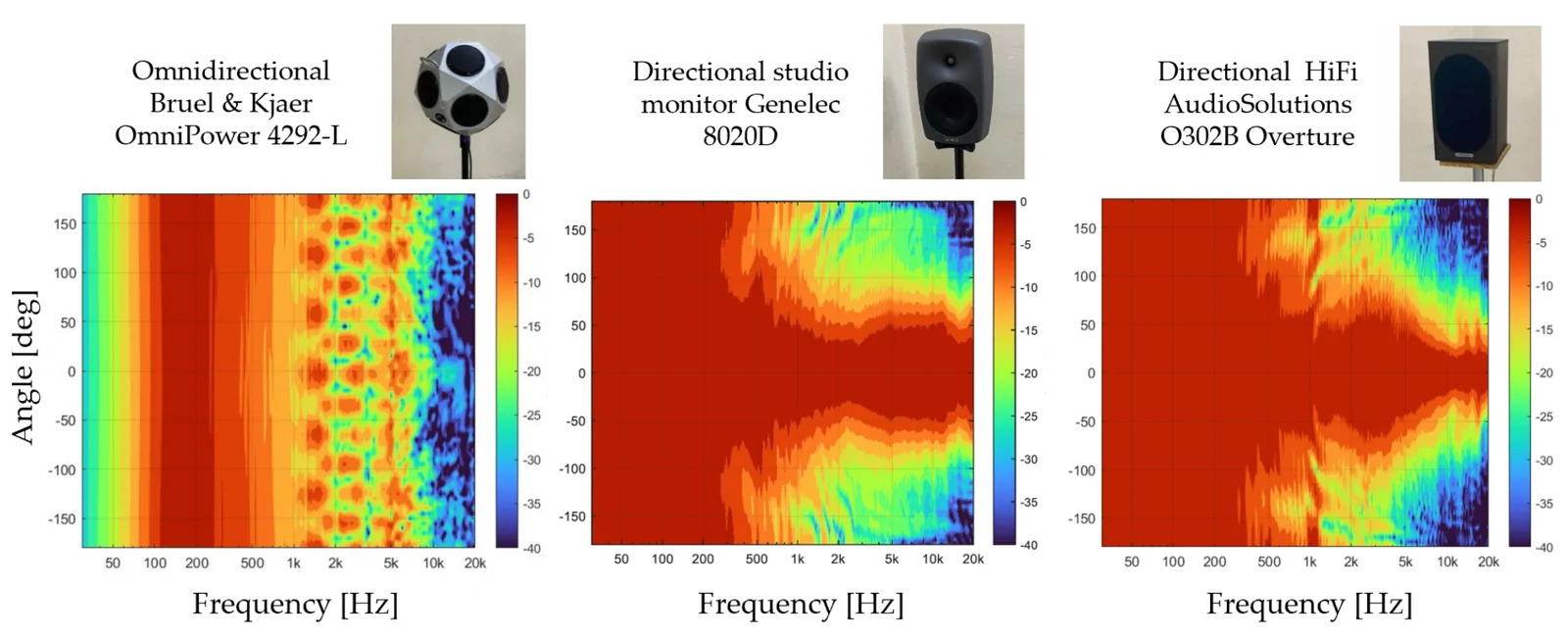
Sound sources used in the experiment and their measured radiation characteristics: Brüel & Kjær OmniPower 4292-L, Genelec 8020D, and AudioSolutions O302B Overture.

The radiation characteristics of all three sources were independently measured using the KLIPPEL Near Field Scanner (NFS) (Dresden, Germany) at AGH University of Krakow. For each source, 3000 spatial measurement points were used, following the measurement procedures recommended by KLIPPEL and the established NFS methodology [36,37]. The horizontal-plane radiation characteristics presented in Figure 4 were generated directly using the KLIPPEL NFS software dB Lab 212, with an angular resolution of $1^{\circ }$. The angle of $0^{\circ }$ corresponds to the acoustic axis of each source, and the displayed sound pressure levels were normalized to the corresponding on-axis response. The same angle–frequency representation and display scale were used for all three devices. The OmniPower source exhibits comparatively broad radiation over a substantial part of the frequency range, whereas the Genelec and AudioSolutions loudspeakers show increasingly directional behavior with increasing frequency.

Previous studies have shown that practical nominally omnidirectional sources depart from ideal omnidirectionality at higher frequencies and that source radiation and orientation can influence measured room impulse responses and derived room-acoustic parameters [15,16,17,20]. In the present work, the NFS measurements provide an independent physical characterisation of the excitation sources.

### 2.4. Room Impulse-Response Measurement Procedure

For every absorber configuration, measurements were carried out for each of the three sound sources, both source positions, the five measurement microphones, and both HATS ear channels. The acquisition procedure and receiver geometry were kept unchanged between configurations so that absorbing-material placement and excitation source remained the principal experimental variables.

Room impulse responses were measured using a swept-sine excitation generated in Room EQ Wizard v5.3 (REW). The sampling frequency was 48 kHz and the duration of each sweep was 10.5 s. The excitation level was calibrated to 90 dB SPL and kept unchanged throughout the measurement campaign. For each measurement condition, four consecutive sweeps were acquired and internally averaged in REW before further room-impulse-response processing. The same acquisition settings were used for all absorber configurations, source types, source positions, and receiver channels.

The swept-sine measurements were recorded and converted to room impulse responses in REW. The resulting impulse responses were then imported into DIRAC 6.0, where the one-third-octave $T_{20}$ and $C_{80}$ values were calculated using the same processing settings for all configurations and sources. The parameter data exported from DIRAC were subsequently processed in MATLAB 2025a for averaging, calculation of paired differences and broad-range summaries, and graphical representation.

Decay-time processing followed the conventions of ISO 3382-2 [38]. Similarly, EBU Tech 3276 was used as a reference for defining the stereo-like source–listener geometry, whereas the ISO framework was used for room-acoustic parameter processing. These references therefore served different methodological purposes within the experiment. This distinction is important because the effect of the excitation source on the measured response constitutes one of the research questions addressed in the present study [18,20].

### 2.5. Acoustic Parameters and Spectral Processing

The final analysis was restricted to decay time $T_{20}$ and music clarity $C_{80}$. Both parameters were evaluated in one-third-octave bands over the frequency range from 63 to 5000 Hz.

The parameter $T_{20}$ was calculated in DIRAC 6.0 from the energy decay curve obtained by backward integration of the band-filtered room impulse response (Schroeder integration). Following ISO 3382-2 [38], a linear regression was fitted to the decay curve over the range from −5 to −25 dB, and the corresponding decay time was extrapolated to 60 dB:

(1) $$T_{20}=-\frac{60}{b},$$

where *b* is the slope of the fitted decay curve expressed in dB/s. All calculations were performed using the standard processing procedure implemented in DIRAC, without additional custom algorithms. The signal-to-noise ratio was checked in DIRAC for the 63-Hz band to confirm sufficient measurement quality at the lower limit of the analysed range. At low frequencies, where the sound field is strongly non-diffuse, $T_{20}$ is interpreted as a frequency-resolved decay descriptor rather than a statistical reverberation constant representative of a diffuse field [4,6].

The two parameters were selected to evaluate complementary aspects of the absorber-placement effect. $T_{20}$ characterizes the decay of sound energy in the room, whereas $C_{80}$ describes the balance between early and late arriving energy, which is particularly relevant to music listening. Their combined analysis therefore allows the influence of absorber placement and excitation-source characteristics to be assessed in terms of both decay behavior and temporal energy distribution. The parameter $C_{80}$ was calculated in DIRAC as

(2) $$C_{80}=10\log_{10}\left(\frac{\int_{0}^{0.08}p^{2}\left(t\right)\;dt}{\int_{0.08}^{\infty }p^{2}\left(t\right)\;dt}\right)\;\mathrm{dB},$$

where p(t) denotes the room impulse response measured at the receiver position. The parameter represents the logarithmic ratio between acoustic energy arriving during the first 80 ms and the energy arriving after 80 ms. In this study, $C_{80}$ is used as an early-to-late energy-ratio descriptor, not as a direct measure of the strength of individual first reflections or as a complete perceptual rating of listening quality.

For each absorber configuration, sound source, and one-third-octave frequency band, the spectral curves presented in the article were obtained as arithmetic means over the two source positions and five G.R.A.S. measurement microphones. Each plotted value therefore represents nominally 10 source-position/microphone measurements for a given source and frequency band. The three source types were processed and presented separately. No averaging across the three source types was used in the primary analysis because source dependence forms an explicit part of the research question.

### 2.6. Data-Reduction and Comparison Strategy

The analysis was organised into predefined comparison families selected to isolate individual placement factors. Configurations W1, W2, and W4 were used to compare treatment of the front wall, rear wall, and a representative lateral-wall arrangement. Configurations W3, W4, and W5 formed a controlled comparison in which the absorber quantity and treated wall family remained unchanged while only the longitudinal position along the lateral walls was varied. Configurations W6, W7, and W8 were used to compare different first-reflection-oriented treatment strategies. The corner configurations W9–W12 were included as a complementary comparison, with their results reported in Table 1 separately for each sound source and frequency range. Additional frequency-resolved $T_{20}$ results for these configurations are presented in Appendix A, including the complete 63–5000 Hz range and a detailed low-frequency comparison over 63–160 Hz.

For each absorber configuration and each sound source, study-specific scalar values of $T_{20}$ and $C_{80}$ were calculated as arithmetic means over the available observations from all 20 one-third-octave bands between 63 and 5000 Hz, both source positions, and the five measurement microphones. Each frequency band was assigned equal weight. These values are descriptive summaries of the experimental dataset and are not standardized single-number room-acoustic ratings. The one-third-octave curves remain the primary evidence.

The frequency ranges used for the analysis were defined before evaluating the results, based on the acoustic characteristics of the investigated room. The estimated Schroeder frequency, calculated from the room volume and untreated-room decay time, was approximately 250 Hz. Accordingly, the 63–250 Hz range was selected to represent the low-frequency region dominated by modal behavior, while 315–1000 Hz and 1250–5000 Hz were used to characterize the low-mid and higher-frequency responses, respectively. The overall 63–5000 Hz mean was retained only as a compact descriptive measure of the treatment effect across the entire investigated frequency range. This subdivision allows the frequency-dependent influence of absorber placement and excitation source to be examined without relying exclusively on a single broadband average [3]. Frequency bands, source positions, and microphones constitute repeated observations within the same physical room and were therefore not treated as statistically independent realizations of different rooms.

Changes relative to the untreated condition were calculated using paired samples. For each source-position, microphone, and frequency-band combination, the value obtained for configuration $W_{i}$ was compared with the corresponding value obtained for W0. The resulting differences were then averaged. The following sign convention was used:

(3) $$\Delta T_{20}=T_{20}\left(W_{i}\right)-T_{20}\left(W_{0}\right),$$

and

(4) $$\Delta C_{80}=C_{80}\left(W_{i}\right)-C_{80}\left(W_{0}\right).$$

Accordingly, negative values of $\Delta T_{20}$ indicate a reduction in decay time relative to the untreated room, whereas positive values of $\Delta C_{80}$ indicate an increase in clarity.

A complementary time-domain analysis was performed using reflectograms for W0 and W7. The final comparison uses source position L and HATS channel 2, corresponding to the ear contralateral to the active source. The reflectograms were obtained from room impulse responses band-pass filtered over 125–5000 Hz. The absolute value of each filtered impulse response was expressed in decibels and independently normalized to the direct-sound amplitude of the corresponding measurement. The time axis was aligned so that t=0 represents the arrival of direct sound. The displayed interval from −1 to 25 ms allows the direct sound and early-reflection structure to be examined. The traces therefore represent reflection amplitudes relative to the direct sound in each measurement.

## 3. Results

### 3.1. Overall Response to Fixed-Amount Absorbing-Material Placement

Table 1 summarizes the study-specific mean values of $T_{20}$ and $C_{80}$ for the untreated condition W0 and all fixed-amount absorber configurations W1–W12 over the full 63–5000 Hz range and the three broad analysis ranges. For the untreated room, the full-band mean $T_{20}$ values were 0.76 s for OmniPower, 0.74 s for Genelec, and 0.75 s for AudioSolutions. The corresponding mean $C_{80}$ values were 7.9, 8.7, and 8.7 dB, respectively.

Across W1–W8, the maximum full-band reduction in $T_{20}$ was approximately 0.33 s, while the maximum increase in $C_{80}$ was approximately 5.9 dB. The corner configurations W9–W12 generally produced smaller full-band changes, with maximum reductions of approximately 0.20 s in $T_{20}$ and increases of up to 3.5 dB in $C_{80}$. Thus, even with absorber quantity and mounting principle fixed, the room-level response varied appreciably with spatial deployment.

The corner configurations W9–W12 are further examined in Appendix A, where the frequency-resolved $T_{20}$ results reveal source-dependent variations and isolated band-specific increases relative to the untreated configuration. These observations highlight the limitations of interpreting treatment effectiveness solely from broadband mean values, particularly in the low-frequency modal region. The full-band means are used only as compact descriptive summaries. The three broad analysis ranges show that the relative ordering of configurations can change with frequency, especially below 1 kHz. This frequency dependence is considered explicitly in the controlled comparisons below rather than being replaced by a single broadband ranking.

### 3.2. Effect of the Treated Room Surface

**Figure 5:**
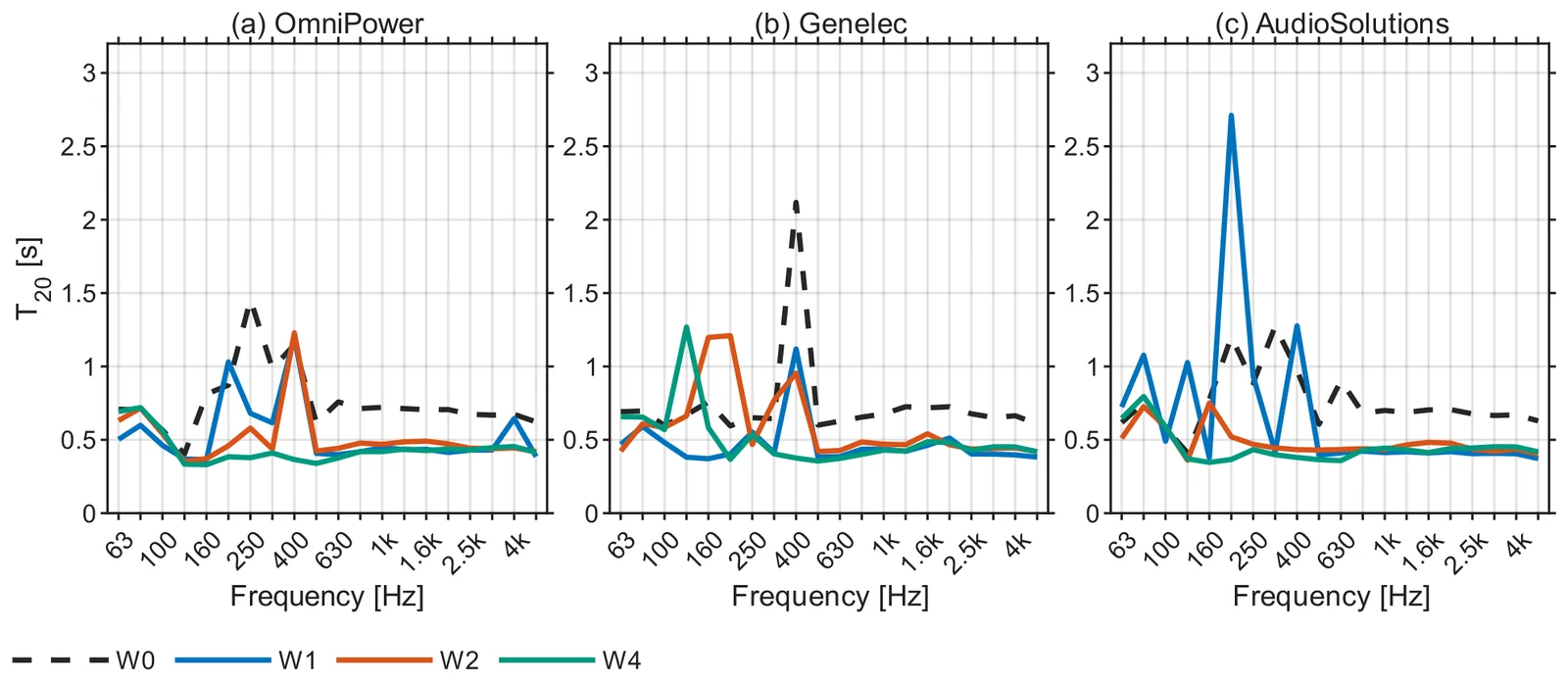
Mean one-third-octave $T_{20}$ for the untreated room W0 and selected front-, rear-, and lateral-wall absorber layouts W1, W2, and W4.

Figure 5 compares $T_{20}$ for W1, W2, and W4 with the untreated condition W0. These configurations isolate three different treatment locations: the front wall (W1), rear wall (W2), and a balanced treatment on the middle part of the two lateral walls (W4). Above the low-frequency region, the three treated responses generally converge and remain below the untreated response. The largest differences among treatment layouts occur in the low and low-mid frequency bands, where the curves also differ substantially between excitation sources.

The five-microphone averages do not identify one $T_{20}$ configuration as lowest for all three sources over the full band: W4 is lowest for OmniPower and AudioSolutions, whereas W1 is lowest for Genelec. The source dependence is most evident below 250 Hz. In contrast, W4 gives the lowest mean $T_{20}$ for all three sources in 315–1000 Hz, while the treated layouts converge more closely in 1250–5000 Hz. Table 1 also shows that W1 gives the highest full-band mean $C_{80}$ within W1/W2/W4 for all three sources.

### 3.3. Longitudinal Position of Treatment on the Lateral Walls

**Figure 6:**
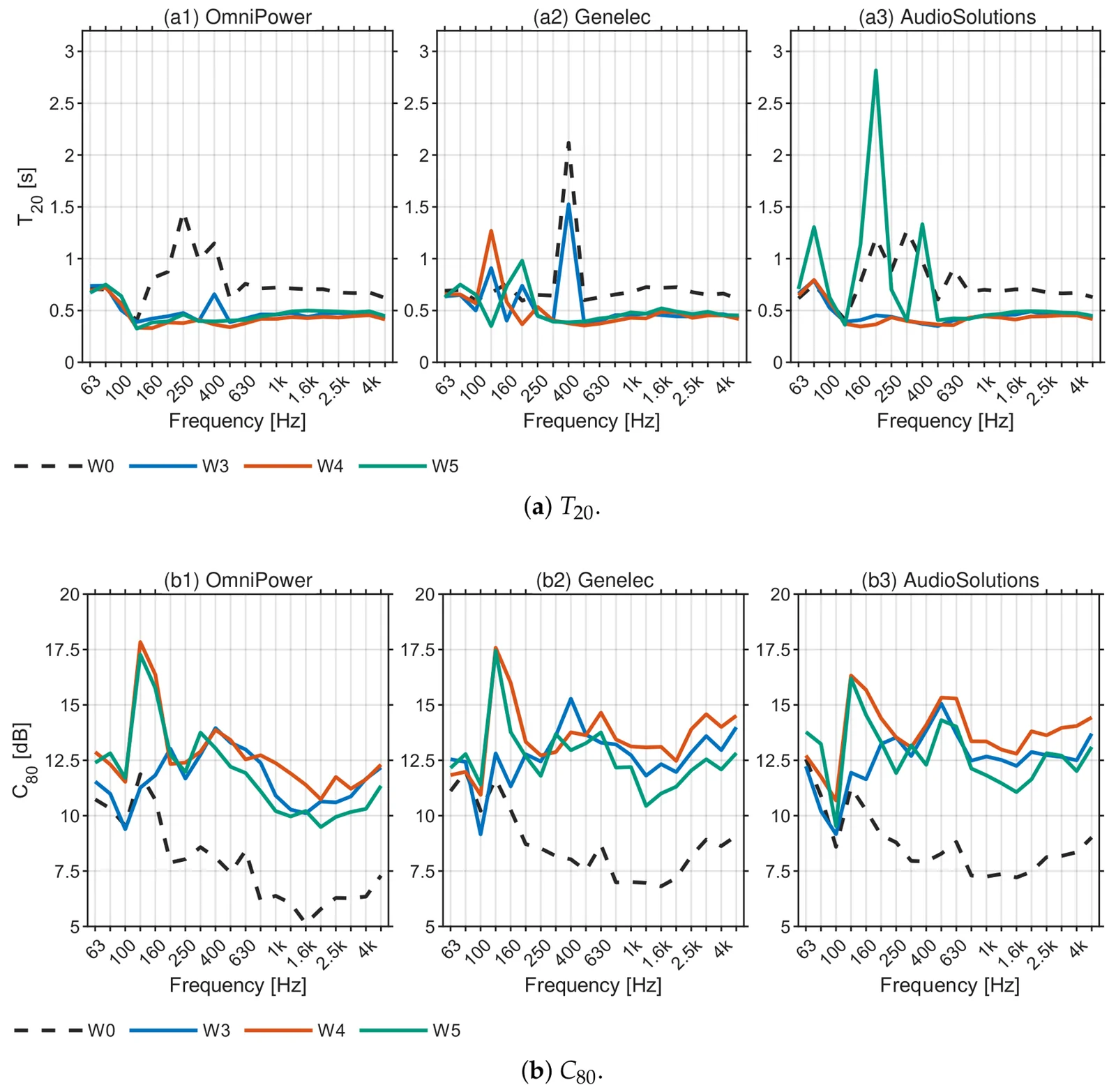
Effect of longitudinal absorber position on the lateral walls for W3, W4, and W5 relative to W0: (**a**) $T_{20}$ and (**b**) $C_{80}$.

The W3–W5 series provides the most tightly controlled placement comparison: absorber quantity and the treated wall family remain unchanged while treatment is moved from the front to the middle and rear parts of the two lateral walls. The corresponding $T_{20}$ and $C_{80}$ results are shown in Figure 6.

Within the controlled W3–W5 comparison, W4 yields the lowest full-band mean $T_{20}$ for all three sources. The same general tendency is observed in the 315–1000 Hz and 1250–5000 Hz ranges, although some source–configuration differences become negligible at the reported precision. The clearest exception occurs in 63–250 Hz, where Genelec gives a lower mean value for W3 than for W4. The low-frequency response is therefore more sensitive to the particular source–placement combination than the higher-frequency response.

A corresponding tendency is visible in $C_{80}$. W4 gives the highest full-band mean clarity within W3–W5 for all three sources and is also highest in most of the frequency-range summaries. For Genelec in the 315–1000 Hz range, W3 and W4 are effectively equal at the reported precision. Thus, the middle lateral-wall placement provides a strong overall treatment tendency within this controlled comparison, while the frequency-resolved data retain source-specific differences that would be obscured by a single broadband mean.

### 3.4. First-Reflection-Oriented Treatment Strategies

**Figure 7:**
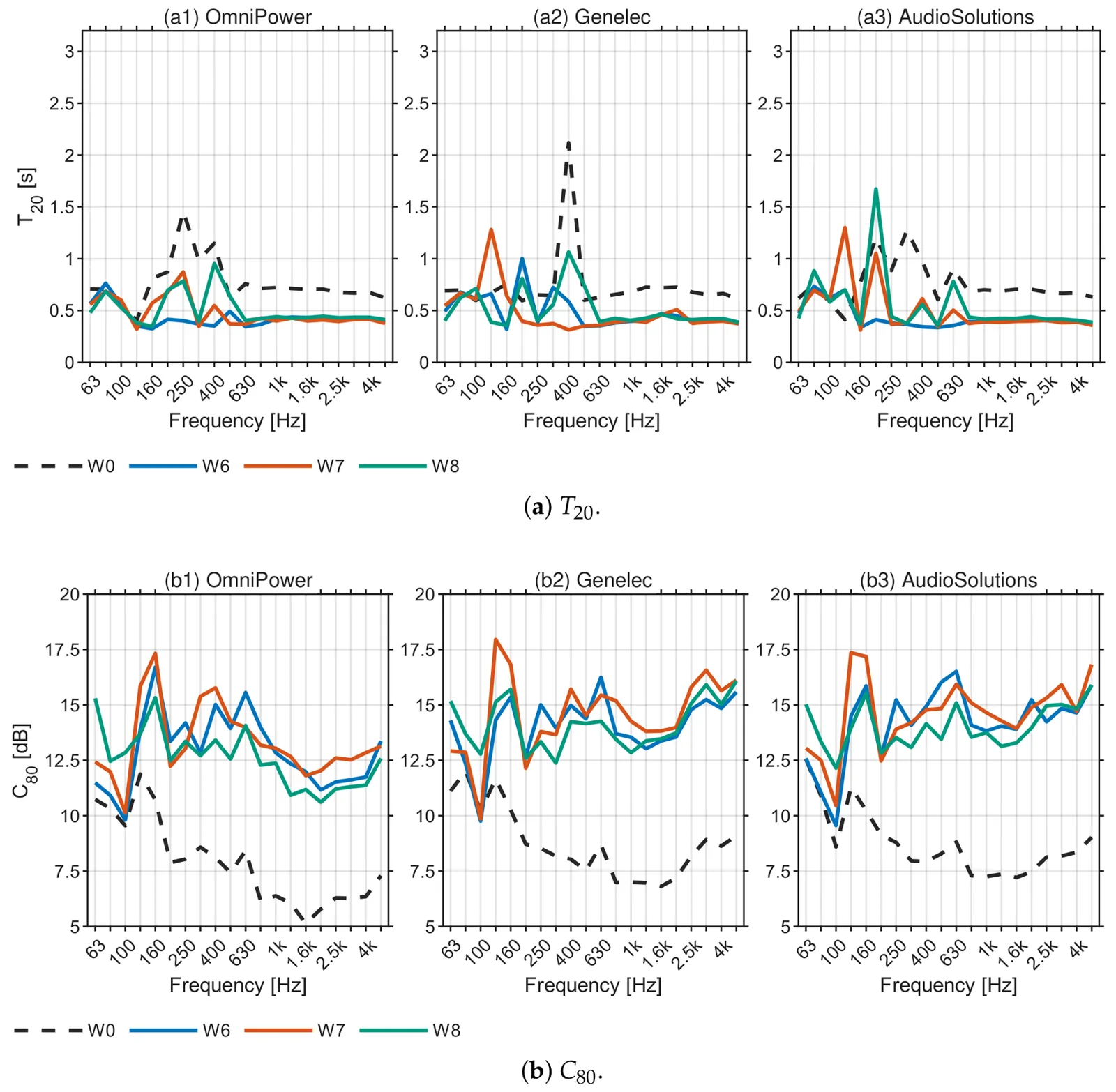
Effect of first-reflection-oriented absorber placement for W6, W7, and W8 relative to W0: (**a**) $T_{20}$ and (**b**) $C_{80}$.

Figure 7 compares W6–W8 with the untreated room. W6 combines the front and lateral first-reflection regions, W7 combines the rear and lateral regions, and W8 combines front- and rear-wall treatment without the corresponding lateral treatment. As in the previous comparisons, the largest differences between treated curves occur mainly in the low and low-mid frequency range, while the higher-frequency responses are more closely grouped.

The five-microphone averages show a source-dependent $T_{20}$ ordering. W6 gives the lowest full-band mean for OmniPower and AudioSolutions, whereas W7 is slightly lower for Genelec. The ordering also changes between the three analysis ranges: W7 becomes the lowest-$T_{20}$ configuration for all three sources only in the 1250–5000 Hz range. This frequency dependence illustrates why the full-band scalar means should be treated as descriptive rather than as universal rankings.

For $C_{80}$, W7 gives the highest full-band mean within W6–W8 for every source and the highest high-frequency mean for all three excitation conditions. In the 315–1000 Hz range it is highest for OmniPower and Genelec and effectively equal to W6 for AudioSolutions at the reported precision. At 63–250 Hz, W8 is higher for OmniPower and Genelec, while W7 and W8 give similar values for AudioSolutions. The relative response therefore remains dependent on both frequency and source.

### 3.5. Time-Domain Analysis of a Representative Source–Receiver Path

**Figure 8:**
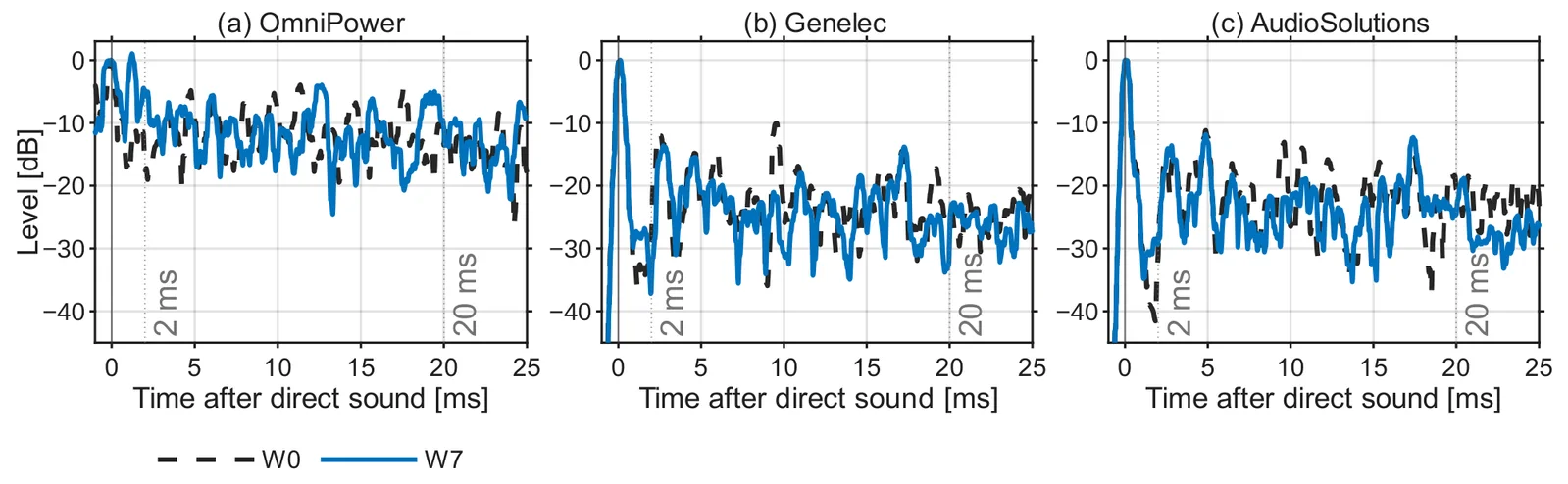
Reflectograms obtained from the absolute values of band-pass-filtered (125–5000 Hz) room impulse responses for W0 and W7 at source position L and contralateral HATS channel 2. Each trace is independently normalized to its direct-sound amplitude.

Figure 8 complements the parameter-domain results with a time-domain comparison of W0 and W7 for source position L and contralateral HATS channel 2. The traces were band-pass filtered over 125–5000 Hz and normalized to the direct-sound reference. The displayed interval from −1 to 25 ms emphasizes the direct sound and early-reflection structure, with guides at 2 and 20 ms.

The W0-to-W7 change in the early response differs between the three sources. For OmniPower, integrated energy between 2 and 20 ms changed by only +0.08 dB, indicating essentially unchanged energy over this interval for the selected path. In contrast, the corresponding changes were −2.04 dB for Genelec and −1.28 dB for AudioSolutions. The difference becomes larger at later times: between 20 and 80 ms the energy changes were −0.99, −3.88, and −3.54 dB for OmniPower, Genelec, and AudioSolutions, respectively, while in the 80–500 ms interval they reached −6.13, −9.06, and −8.85 dB.

For the same representative path, the corresponding $C_{80}$ changes from W0 to W7 were +6.34 dB for OmniPower, +7.86 dB for Genelec, and +7.63 dB for AudioSolutions. The time-domain response therefore shows that W7 does not act as a uniform attenuation of the first 20 ms. Instead, modification of the early-energy region is source dependent, whereas later energy is reduced more strongly for all three sources. This behavior provides direct temporal context for the source-dependent $C_{80}$ changes observed in the frequency-domain analysis.

## 4. Discussion

### 4.1. Placement and Installed Performance of the Absorbing Material

The principal finding is that a fixed amount of the same absorbing material does not produce a fixed room-level effect when its spatial deployment is changed. The controlled W3–W5 comparison demonstrates this directly because material quantity, wall family, and mounting condition were unchanged while only the longitudinal position on the lateral walls was varied. Within this comparison, W4 provided the lowest full-band mean $T_{20}$ and the highest mean $C_{80}$ for all three sound sources. This favorable performance may be partly explained by the location of the absorbers in the middle sections of the lateral walls, which partially overlap with the geometrically determined first-reflection regions for the fixed source–listener arrangement. Such placement may provide more effective interaction with dominant reflection paths while distributing absorption symmetrically across both lateral boundaries. Frequency-dependent coupling to the room’s modal sound field may also contribute to the observed differences, particularly at low frequencies. However, W4 should not be considered a globally optimal configuration, since other arrangements, particularly W6 and W7, achieved better results for selected parameters and excitation sources. The first-reflection configurations provide the same general conclusion for a second placement family: equal absorber area can produce different decay and clarity responses depending on which propagation regions are treated.

This behavior is consistent with earlier absorber-placement studies showing that room-level effectiveness depends on where absorbing material is exposed to the sound field, rather than on treated area alone [2,8,9,10,11,12]. The present experiment extends that principle to a full-scale listening room with a fixed stereo-like source–listener geometry. In such a geometry, front, rear, lateral, and first-reflection regions do not carry equivalent acoustic energy or serve equivalent propagation paths. The smaller full-band changes generally observed for W9–W12 should therefore be interpreted only within the tested 100-mm porous-foam arrangements; they do not imply that corner treatment is generally ineffective.

From a materials perspective, the distinction is important. Relocating the absorbers changes how the room field interacts with the installed material and therefore its effective in-room utilization. This is consistent with the broader distinction between laboratory material characterization and in situ room performance [1,2,39,40]. Material properties and laboratory absorption data describe the absorber itself, whereas the realized room-level effect additionally depends on the installation configuration.

### 4.2. Frequency Dependence and Source–Placement Relationship

The placement effect is strongly frequency dependent. Across the spectral figures, the largest configuration-to-configuration differences occur mainly at low and low-mid frequencies, whereas treated responses become more similar at higher frequencies. This is compatible with the physics of small rooms, where low-frequency decay is governed by a limited number of resonant contributions whose excitation and damping depend on source position, receiver position, and boundary conditions [3,4,6,7].

The revised range-based summaries also show why a single 63–5000 Hz mean cannot fully describe treatment performance. For the treated-surface comparison, W4 was the lowest-$T_{20}$ layout for all sources in 315–1000 Hz, but the 63–250 Hz ordering depended on source. In the first-reflection comparison, W7 gave the lowest high-frequency $T_{20}$ for all three sources, while the low- and mid-frequency rankings differed. Similar source-specific changes appear in $C_{80}$. These results show that the source effect is not a simple constant offset; it modifies the relative response of the placement strategies as a function of frequency.

The measured radiation characteristics in Figure 4 provide a physical context for this observation. The quasi-omnidirectional source and the two playback loudspeakers distribute acoustic energy differently with angle and frequency, so a treatment located on a particular surface need not intercept the same fraction of radiated and reflected energy for all three sources. This interpretation is consistent with previous work showing that source radiation and orientation affect measured room impulse responses and derived parameters [15,16,17,20,41,42].

The reflectogram comparison supports the same interpretation in the time domain. For the representative W0–W7 path, energy in the 2–20 ms interval was essentially unchanged for OmniPower but was reduced for Genelec and AudioSolutions, whereas 80–500 ms energy was reduced much more strongly for all three sources. The treatment therefore changes the early/late energy distribution in a source-dependent manner rather than acting as uniform broadband attenuation.

### 4.3. Implications for Listening-Room Treatment Validation

The results have a practical consequence for acoustic-treatment validation. A quasi-omnidirectional source remains appropriate for standardized room characterization and provides a repeatable excitation condition; the present study does not argue otherwise. It does show, however, that the apparent effect of a treatment in a small listening room can differ when the same room is excited by the playback loudspeakers for which the room is functionally intended.

This distinction is compatible with the different roles of ISO 3382-2 and EBU Tech 3276. ISO-based definitions and processing provide a framework for room-acoustic decay measurements, whereas EBU Tech 3276 defines conditions relevant to critical listening and monitor loudspeakers [21,38]. Neither is a dedicated protocol for quantifying a source × absorber-placement interaction. For practical validation of treatment in a studio or listening room, it is therefore useful to report the excitation source explicitly and, where relevant, to complement measurement-source characterization with the intended playback loudspeaker or a source with comparable radiation behavior.

The same argument reinforces the value of frequency-resolved reporting. Broadband descriptive means are useful for establishing the overall scale of a treatment effect, but the present data show that configuration ordering can change between low, mid, and high frequency ranges. Design conclusions should therefore be tied to a stated metric, source, and frequency context rather than to a single universal ranking of treatment layouts.

### 4.4. Implications of Excitation-Source Dependence for Acoustic Treatment Design

The observed excitation-source dependence has direct implications for the design of acoustic treatment in small listening rooms. In practical room-acoustic work, the treatment strategy is often developed and assessed using a measurement-oriented quasi-omnidirectional source, while the room is ultimately operated with directional playback loudspeakers. The present results show that these two excitation conditions cannot be assumed to produce the same apparent treatment effect. For the same absorber configuration, the measured $T_{20}$ and $C_{80}$ values changed with source type, and the relative ordering of configurations also varied with frequency. The source effect should therefore not be interpreted as a simple constant offset applied to an otherwise unchanged room response.

This distinction is important when acoustic treatment is designed toward a target decay or clarity condition. If the design process relies exclusively on measurements obtained with a quasi-omnidirectional source, the treatment may be optimized for an excitation field that differs from the one produced during normal room operation. When the room is subsequently used with directional loudspeakers, the realized response may therefore be either more strongly or less strongly damped than suggested by the measurement-source condition. In practical terms, this creates a risk of either excessive or insufficient treatment relative to the intended operational condition, particularly in frequency regions where the interaction between source radiation and absorber placement is strongest.

The present results therefore support treating the sound source as part of the acoustic-design problem rather than only as a measurement tool. Standardized or quasi-omnidirectional excitation remains valuable for repeatable room characterization, but for rooms intended for critical listening, the final treatment strategy should also consider the radiation characteristics of the playback system for which the room is being designed. In this sense, the realized performance of an acoustic treatment is determined not only by the absorber properties and installed area, but by the combined interaction between material placement, room geometry, source–listener geometry, frequency range, and excitation-source characteristics. A room that satisfies a target acoustic condition under one excitation source should therefore not automatically be assumed to provide an equivalent response under another source-radiation condition.

### 4.5. Limitations

The experiment was performed in one full-scale room using one porous absorber type and three excitation devices. The results therefore identify controlled trends within this room rather than a universally optimal arrangement for all listening rooms. The three sources differ in more than radiation directivity, so the experiment demonstrates a source–placement dependence but does not isolate directivity as an independent causal variable. The low-frequency $T_{20}$ values also require the modal-field interpretation described above, and the descriptive means should not be treated as standardized single-number ratings.

The primary spectral analysis averages two source positions and five free-field microphones within the same physical room. These observations are repeated spatial samples rather than statistically independent rooms, so they were not used for inferential ranking. Independent re-installation repeatability of each absorber layout was not evaluated. Under the fixed source–receiver geometry and room-acoustic positioning tolerances used here, small setup-repositioning effects were treated as secondary to the deliberately larger changes in treatment location, although a separate installation-repeatability uncertainty is therefore not available. The HATS data were intentionally removed from the primary averages and retained only for the representative reflectogram because binaural ear channels apply a different directional weighting to arriving sound. Broader transferability would require additional rooms, absorber systems, and source geometries. Within these limits, the central conclusion remains that installed absorbing-material performance depends jointly on spatial deployment and excitation conditions.

## 5. Conclusions

This study investigated how the spatial deployment of a fixed amount of the same sound-absorbing material affects the acoustic response of a full-scale small listening room and how the measured effect depends on the excitation source. Using five free-field measurement microphones for the primary analysis, the results show that changing absorber location while keeping material type, quantity, and mounting principle constant produces substantial and frequency-dependent differences in both $T_{20}$ and $C_{80}$.

The controlled comparisons demonstrate that treatment performance depends on the relationship between material placement and the fixed source–listener geometry. The lateral-wall series produced a strong overall tendency for the middle placement W4 to reduce decay and increase clarity, but the detailed low-frequency results still contained source-specific exceptions. The first-reflection comparison showed an even clearer dependence on source and frequency: no single configuration produced the lowest $T_{20}$ in every range and for every source, although W7 produced the highest full-band mean $C_{80}$ within W6–W8 for all three excitation conditions.

The measured placement effect was therefore source dependent rather than a fixed property of the absorber layout. The time-domain comparison supported this conclusion by showing different changes in early reflected energy for the quasi-omnidirectional source and the two playback loudspeakers, while later energy was reduced more consistently. These differences are consistent with the measured source radiation characteristics, but they cannot be attributed to directivity alone because the devices also differ in other electroacoustic properties.

The broader implication is that laboratory material characterization and installed absorber area are not sufficient to predict treatment performance in a small listening room. Material properties, laboratory absorption data, spatial deployment, source–listener geometry, frequency range, and excitation conditions should be considered together. The present results do not define a universally optimal absorber arrangement; instead, they show that the effectiveness of an installed absorbing material is configuration-, frequency-, metric-, and source-dependent.

## Figures and Tables

**Table 1:** Descriptive mean values of $T_{20}$ and $C_{80}$ for the untreated condition W0 and fixed-amount absorber configurations W1–W12.

Variant	Source	$T_{20}$ [s]				$C_{80}$ [dB]			
		63–5000	63–250	315–1000	1250–5000	63–5000	63–250	315–1000	1250–5000
W0	OmniPower	0.76	0.79	0.82	0.68	7.9	9.9	7.5	6.2
	Genelec	0.74	0.67	0.89	0.68	8.7	10.4	7.7	8.0
	AudioSolutions	0.75	0.75	0.86	0.68	8.7	10.2	7.9	8.0
W1	OmniPower	0.53	0.57	0.58	0.45	13.1	14.3	13.2	11.9
	Genelec	0.47	0.46	0.53	0.43	14.2	14.6	13.7	14.4
	AudioSolutions	0.68	1.05	0.56	0.41	14.0	13.8	13.7	14.5
W2	OmniPower	0.52	0.52	0.58	0.46	12.3	13.9	11.8	11.0
	Genelec	0.60	0.74	0.59	0.46	13.1	13.4	12.2	13.5
	AudioSolutions	0.48	0.56	0.44	0.45	13.2	13.4	12.2	14.0
W3	OmniPower	0.49	0.53	0.47	0.46	11.6	11.4	12.7	10.9
	Genelec	0.55	0.61	0.60	0.45	12.7	11.9	13.6	12.8
	AudioSolutions	0.47	0.52	0.40	0.46	12.6	11.7	13.4	12.7
W4	OmniPower	0.44	0.49	0.39	0.44	12.7	13.6	13.0	11.6
	Genelec	0.51	0.66	0.39	0.45	13.6	13.5	13.6	13.7
	AudioSolutions	0.45	0.51	0.40	0.44	13.8	13.6	14.1	13.7
W5	OmniPower	0.48	0.52	0.42	0.49	11.9	13.5	12.0	10.2
	Genelec	0.52	0.65	0.42	0.48	12.6	13.1	13.0	11.7
	AudioSolutions	0.72	1.09	0.57	0.48	12.8	13.2	13.0	12.1
W6	OmniPower	0.43	0.48	0.39	0.42	12.9	12.9	14.0	12.0
	Genelec	0.49	0.59	0.46	0.42	14.0	13.4	14.5	14.3
	AudioSolutions	0.43	0.52	0.36	0.40	14.2	13.1	14.9	14.7
W7	OmniPower	0.48	0.61	0.41	0.41	13.3	13.2	14.3	12.5
	Genelec	0.48	0.64	0.36	0.41	14.5	13.8	14.8	15.1
	AudioSolutions	0.51	0.69	0.43	0.39	14.6	13.8	14.9	15.1
W8	OmniPower	0.51	0.56	0.54	0.43	12.6	13.6	12.9	11.3
	Genelec	0.51	0.53	0.60	0.42	14.1	14.1	13.6	14.7
	AudioSolutions	0.55	0.72	0.49	0.42	14.0	13.8	13.8	14.4
W9	OmniPower	0.56	0.63	0.56	0.50	11.1	12.1	11.4	9.9
	Genelec	0.57	0.57	0.65	0.50	12.0	12.7	11.6	11.5
	AudioSolutions	0.67	0.93	0.57	0.50	11.9	11.8	12.2	11.6
W10	OmniPower	0.69	0.83	0.76	0.50	11.2	13.2	10.9	9.5
	Genelec	0.61	0.83	0.48	0.50	12.0	13.2	11.2	11.4
	AudioSolutions	0.61	0.80	0.52	0.50	11.9	12.6	11.3	11.6
W11	OmniPower	0.61	0.72	0.61	0.51	10.7	11.4	11.1	9.6
	Genelec	0.58	0.54	0.72	0.50	11.6	11.7	11.6	11.4
	AudioSolutions	0.58	0.79	0.44	0.50	11.7	11.7	11.9	11.5
W12	OmniPower	0.61	0.61	0.74	0.51	11.1	13.0	10.9	9.5
	Genelec	0.64	0.71	0.69	0.52	11.8	12.8	11.4	11.1
	AudioSolutions	0.69	0.96	0.61	0.50	12.3	13.1	11.9	11.7

**Figure A1:**
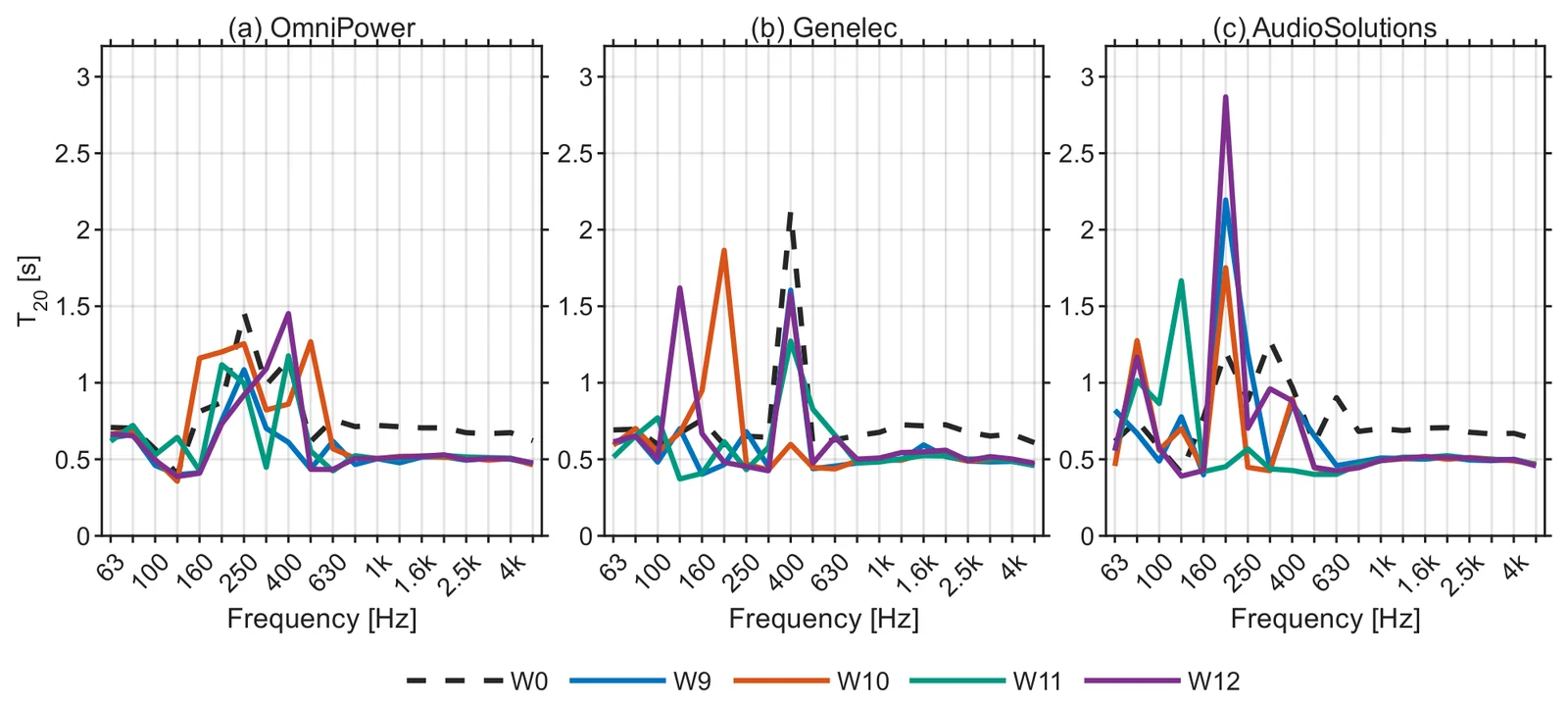
Mean one-third-octave $T_{20}$ values for the untreated configuration W0 and corner absorber configurations W9–W12 over 63–5000 Hz: (**a**) OmniPower, (**b**) Genelec, and (**c**) AudioSolutions. Each curve represents the mean over two source positions and five measurement microphones.

**Figure A2:**
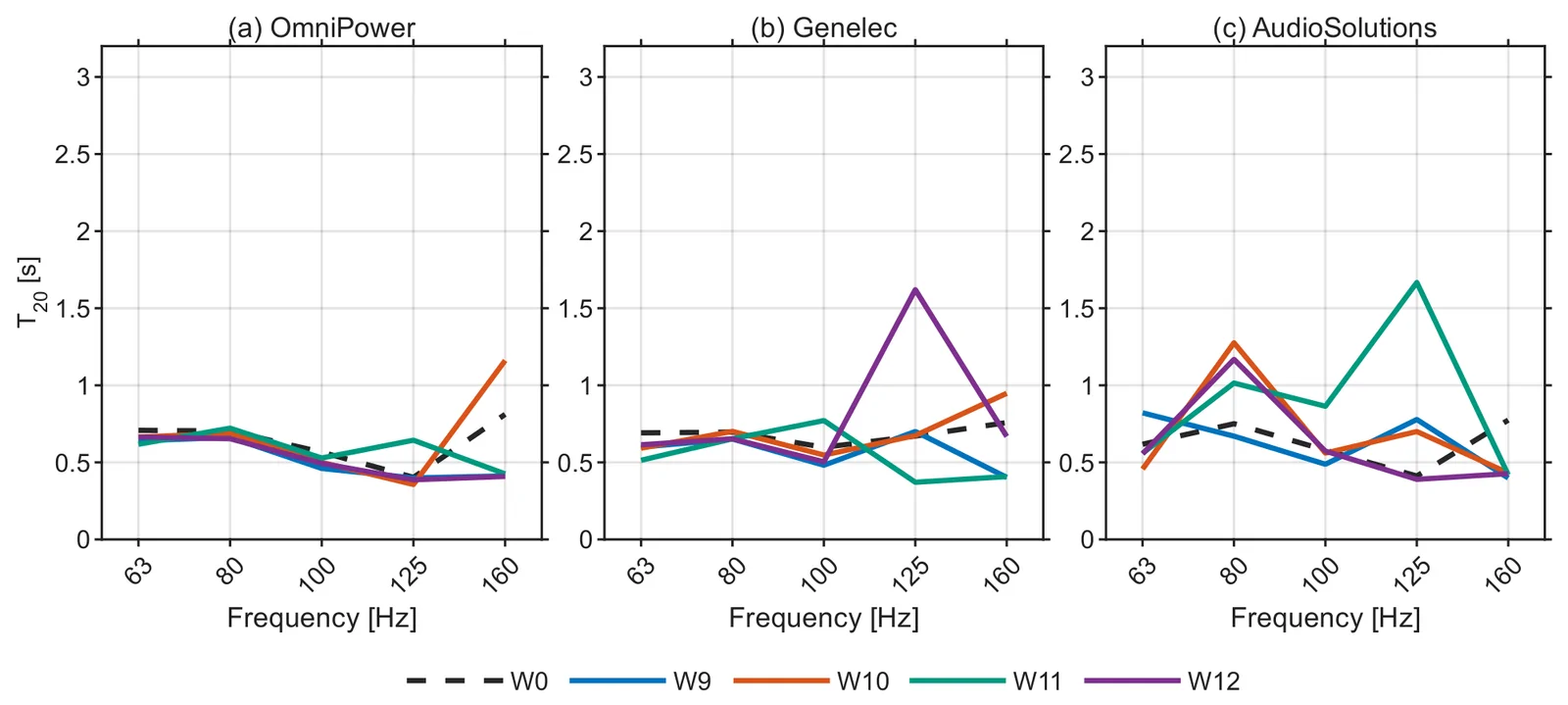
Low-frequency detail of the $T_{20}$ results for W0 and W9–W12, covering the one-third-octave centre frequencies from 63 to 160 Hz. The source order, configurations, and averaging procedure are identical to those in Figure A1.

## Data Availability

The original contributions presented in this study are included in the article. Further inquiries can be directed to the corresponding author.

## Appendix A. Frequency-Resolved Decay-Time Results for Corner Absorber Configurations

This appendix provides additional frequency-resolved $T_{20}$ results for the corner absorber configurations W9–W12, complementing the descriptive values reported in Table 1. All four configurations used 12 identical polyurethane-foam absorbers with a total nominal area of $6\;m^{2}$. The treatment locations were the two front corners (W9), two rear corners (W10), diagonally opposite front and rear corners (W11), and front and rear corners on the same side of the room (W12). The untreated configuration W0 was retained as a reference.

The $T_{20}$ values were evaluated in one-third-octave bands using the same measurement and processing procedures as in the main study. Each spectral curve represents the arithmetic mean over the two nominal source positions and five free-field measurement microphones, calculated separately for the OmniPower, Genelec, and AudioSolutions sources. Figure A1 presents the complete 63–5000 Hz range, whereas Figure A2 provides a detailed comparison over 63–160 Hz, where pronounced configuration-dependent variations are observed.

The results demonstrate a strongly frequency-dependent response to corner treatment. Although all four corner configurations yielded lower full-band mean $T_{20}$ values than the untreated room for each excitation source (Table 1), this improvement was not uniform across individual frequency bands. At medium and higher frequencies, the treated responses generally converge toward similar decay-time values. In contrast, pronounced differences and isolated increases in $T_{20}$ relative to W0 are observed in the low-frequency and transition regions.

The frequency-range averages in Table 1 confirm that this non-monotonic behavior is not restricted to individual spectral peaks. For example, in the 63–250 Hz range, configuration W10 yielded a mean $T_{20}$ of 0.83 s for OmniPower, compared with 0.79 s for W0. For AudioSolutions, configuration W12 yielded 0.96 s compared with 0.75 s for the untreated room. The magnitude and frequency of such differences depend on both absorber placement and excitation source.

An increase in the measured $T_{20}$ after the installation of absorbing material should not necessarily be interpreted as an increase in the overall acoustic energy or as evidence that the absorbers introduce negative damping. In the non-diffuse low-frequency sound field of a small room, the decay observed within a one-third-octave band can contain contributions from several resonant modes with different decay rates. Redistributing or introducing absorbing material may modify the relative contributions and damping of these modes, changing the shape of the band-limited energy decay curve. Consequently, the $T_{20}$ value obtained from a linear regression over the −5 to −25 dB decay range may increase even when the overall room response benefits from additional absorption [4,6].

The differences between excitation sources provide an additional indication of the source-dependent nature of the measured response. Source radiation characteristics influence the distribution of acoustic energy in the room and may alter the relative excitation of modes contributing to the measured decay. Accordingly, the same corner treatment can produce different band-specific $T_{20}$ values for the quasi-omnidirectional source and the directional playback loudspeakers. Nevertheless, the observed differences cannot be attributed exclusively to directivity, since the sources also differ in their other electroacoustic characteristics and the low-frequency field is strongly modal.

These supplementary results therefore reinforce the principal finding of the study: the measured effectiveness of a fixed amount of absorbing material depends on its spatial distribution, the frequency range considered, and the excitation conditions. The isolated increases in band-limited $T_{20}$ should be interpreted cautiously, particularly in the modal region, and do not establish that corner treatment is generally detrimental. A detailed identification of the contributing modal decay rates would require an additional modal analysis beyond the scope of the present study.
